# Access to Care During a Global Health Crisis

**DOI:** 10.1089/heq.2020.29001.rtl2

**Published:** 2020-05-06

**Authors:** Debra Furr-Holden, Olivia Carter-Pokras, Mary Kimmel, Charles Mouton

**Affiliations:** ^1^ Division of Public Health, Michigan State University, Flint, Michigan, USA.; ^2^ Department of Epidemiology and Biostatistics, University of Maryland School of Public Health, College Park, Maryland, USA.; ^3^ Latino Health Steering Committee, Montgomery County, Silver Spring, Maryland, USA.; ^4^ North Carolina Maternal Mental Health MATTERS, North Carolina, USA.; ^5^ University of North Carolina, Perinatal Psychiatry Program.; ^6^ Department of Psychiatry, University of North Carolina, North Carolina, USA.; ^7^ School of Medicine, University of Texas Medical Branch at Galveston, Galveston, Texas, USA.

## Introduction

Access to care has been an ongoing health care issue for socially and economically disadvantaged populations in the United States for many decades. The recent COVID-19 pandemic has highlighted these disparities as people of color suffer disparate mortality and face growing inequities in care. This moderated panel discussion provides a broad insight into these issues and discusses the need for greater attention to the access to care problem for many U.S. communities.

***DR. DEBRA FURR-HOLDEN:* Thank you all for joining on this roundtable discussion about health equity and access to care. To start our conversation, I would love to hear from each of you about what you see as the current highest priorities regarding access to care.**

**DR. OLIVIA CARTER-POKRAS:** In preparation for this discussion, I reached out to other members of the Latino Health Steering Committee from Montgomery County, Maryland, because we are a mix of academics as well as health care providers and others, so I wanted to bring in voices other than just my own.^1^ At the time of this discussion, we are dealing with the COVID-19 pandemic.

There are many factors that can result in lower access and quality of care received by racial and ethnic minorities, in general, and specifically for those with COVID-19 infection.^2–4^ These factors can include not knowing where to go for testing or where to get care, for instance, as well as the ability to pay, transportation to get there, and whether or not the person has a usual health care provider (referral is often required to receive testing). The lack of interpretive services continues to be a big issue. Only about half of foreign-born Latinos in Maryland have health insurance, even though Maryland is ranked second in the nation in median household income.^5,6^ Health issues are interrelated with other problems such as unemployment and stress. These are long-standing concerns that have only been exacerbated by the coronavirus pandemic.

On top of that, we have individuals who are undocumented and afraid to seek care. As we learned after 9/11, many are “frightened because we don't know what is going to happen (with immigration)…this is an emergency.”^7^ That fear certainly is coming into play with people who, even if they have the signs and symptoms, even though they are very ill, are refusing to go in for care. Many of them were afraid to leave their homes even before all of this happened because of that fear.

Immigrant workers are a significant share of essential workers these days, from staffing grocery stores to cleaning hospital rooms and transporting food.^8^ Although 17% of civilians working in 2018 were foreign born, it is a much larger percentage when you look specifically at workers on the front line.

Immigrant workers are also overrepresented in jobs that are currently shuttered, such as hotels and restaurants, cleaning services, personal services, and hair and nail care. Lack of income right now is a big issue and a major disparity during this crisis.

Language is still an issue when trying to figure out where to go for testing; information on testing has mainly been available only in English.^9^

And finally, inconsistent messaging continues to be a major challenge. I think we learned a lot in the past about not sufficiently dealing with health literacy.^10^ When we think about who should get tested, we get one message from the CDC, one message from the state, and one message from the county health department, but none of them are consistent, and, frankly, misinformation within our communities has been shared by the mainstream media as well as through social media. For instance, using Neosporin in the nose to help prevent getting the virus was one piece of misinformation that was spread early on.^11^

These issues existed before COVID-19, such as the lack of insurance and the lack of interpretation and translation of materials, but the pandemic has compounded the problem significantly.

**DR. MARY KIMMEL:** I want to talk about mental health priorities regarding access to care. Building upon what Dr. Carter-Pokras just talked about, the same stressors are still there, and the same issues are still there, but now things are magnified. I actually hope that one silver lining would be that we can learn from these issues and figure out how to handle some of them. We are seeing that with mental health.

We already had many people who did not feel able to talk about their mental health or mental wellness. And I like the term “mental wellness,” because when I say “mental health,” people often think of that as illness. I really want us to start thinking about mental health and wellness as our mental life, which includes for all us all the things we manage, our relationships, the stress we manage such as from limited finances and access to food and housing and health care.

So those things were already there, and people already did not feel they could always talk about how they were feeling and their emotions and how they were managing stressors and afraid what people would think if they did talk about how they are managing stressors.

But now, I think the good thing about this crisis is that people are acknowledging that this is highly stressful and that this is something that is going to make other people very stressed too. Going forward, I anticipate that we are only going to see increases of this reflection on some of the things in the news and thinking about the number of people who are being impacted by this in different ways, either by family members being ill, being afraid that they are ill and not knowing, not knowing whether they are going to infect their families, and just the trauma of all this is going to be longstanding.

One nice thing is this has really propelled us forward in terms of telehealth. I am the medical director for a program called North Carolina Maternal Mental Health MATTERS, and that program provides education to frontline providers, such as obstetricians and family practice physicians and pediatricians, to help them support mental health in their patients.

But through that, we also had intended to pilot a telehealth assessment that we could do to support primary care providers with psychiatric assessment. Owing to new support for telehealth, we have been able to move forward with these telehealth assessments. Whereas this was previously taking a while to get going, are now being pushed forward, and payers are now saying this is something they are going to pay for.

Going forward, we need to work to say that those things are important, and we should continue those things even after the immediate need is gone.

But I also, from that, have found some of the inequity. I certainly have worked with patients who are supposed to be doing our visits through our medical records system but are experiencing access challenges. Some patients cannot access the Internet, and they do not have the bandwidth to do that kind of visit. I have done phone visits with some patients, and I really hope that we can also move that forward as an option when video visits for mental health support are not possible. That has historically been something that has not been compensated as well, to do phone visits, and I really hope that is something now that payers will start to think about as important. The telephone visits have gone very well. But even there, again, a patient may experience inequity, because there are some patients who do not have a reliable phone or a secure private place to talk. This is particularly true as families are being forced to stay home. So still some patients are not able to talk as openly about their mental health needs and about stress they are facing if they cannot find privacy. For example, this is certainly relevant as we are seeing the increases in intimate partner violence some individuals are facing in their “shelter-in-place” housing situations.

So, by moving all these things to telehealth, I think we have to be cognizant that some patients are not going to be able to access those things, and so in some ways, we are taking away barriers from people getting access to mental health care, but I also think we are also highlighting some increased barriers for some individuals.

**DR. CHARLES MOUTON:** Yes. Thank you. I think that some of the things that Dr. Kimmel has mentioned are also true in primary care. It looks like this health crisis is going to usher in a new way to approach health care and primary care, but I think in a lot of respects, these possible advancements leave behind some of the most vulnerable. With the COVID-19 crisis, there have been closures of many primary care clinics, federally qualified health centers, and urgent care centers, with many of them switching to telemedicine visits.

The issues highlight that many of the most needy do not have access to appropriate Internet or computers to do any kind of video visits; this applies in the primary care setting as well. The inability to get adequately reimbursed for telehealth visits, with the requirement that it must be video captured, means that these telehealth visits may not get reimbursed. These are things that the Centers for Medicare & Medicaid Services (CMS) is going to need to change if telehealth will move forward.

The impact of the CMS regulations on telehealth for some of the community providers that are not affiliated with large provider networks is even more profound; these are essentially private practices. So now you put them in a position where they do not have the necessary protective equipment to allow patients to come through their doors, they cannot see their patients face to face, and you force them to employ a new telehealth capability that they have to now get up and running. These limitations and loss of patient care revenue have many looking at closure of their practices. So now an already distressed underserved population becomes even more underserved and facing a real stressful time in terms of trying to get access to health care.

In this access to care vacuum, you are seeing people trying to take care of themselves and family members with home remedies. We have heard about the unfortunate incidents with people using hydroxychloroquine inappropriately. We have instances of people, because of the misinformation that is being spread, using Neosporin and other things. I think that people are becoming a little bit more desperate because they are unable to get the care that they feel that they need.

On the opposite end of access to care is the issue, when they do wind up getting hospitalized, they then face problems with the distribution of the current health care resources. This reflects my concern about potential inequities that may arise when the decision has to be made about who gets a ventilator and who does not.

We already know that implicit bias influences health care decision making. We have shown that over and over again. How does implicit bias influence a critical resource decision for people who are the most vulnerable? That question has yet to be explored and adequately addressed.

I do not know of any effective strategies for eliminating implicit bias. And now we are faced with trying to come up with a strategy to deal with critical lifesaving decisions in stressed health care environments. Will these biases have an untoward influence on not only access to care, but also people's general willingness to seek care? These are areas we need to have some attention paid, in particular, to alleviate.

***DR. FURR-HOLDEN:* I have been talking to a colleague who is a physician, and he said he had an “aha moment.” He is an anesthesiologist, so he is there when patients are being intubated, et cetera.**

**And he said he watched a provider have a conversation with a family that said, “This person has a zero percent chance of survival on a vent.” So, you know, in essence, he talked the family into a “Do Not Resuscitate” (DNR). There was really no medical foundation for that.**

**So you talk about implicit bias, and it sounded a little bit like you were talking about bias on the side of the patients. What about bias on the side of providers? You know, we have been looking at and we have been experiencing these tremendous disparities.**

**What we now know and are seeing is that it is not just the disparity of people dying on the vent, but also what is happening in the system of care where they are not even making it to the vent? How do you all think implicit bias is playing into the conversations that have no criteria for anybody else in the room? So some well-meaning provider potentially is steering black and brown and colored and other disparate populations toward DNRs. What do you think about that?**

**DR. MOUTON:** I think that implicit bias is, as they say, a real and present danger. I think providers are well meaning, but they are still making judgments. When thinking about people's ability to recover when they have comorbid conditions, it is important to bear in mind that those comorbid conditions are often a reflection of social inequities and society. And now, providers are having to tell these patients with certain comorbidities that when they need a health care resource, they may be ineligible. Is that compounding the inequities that already exist?

I think it is a difficult question. Ethicists continue to debate the proper distribution of resources during times of scarcity.^12^ But, again, I am convinced that our own implicit biases influence how we make judgments and decisions. I worry that these biases will potentially lead to greater inequities as this crisis moves forward. More importantly, some of the policies and processes that are being put in place to deal with resource scarcity caused by the COVID-19 crisis may wind up becoming the new standard that we turn to when making these kinds of decisions, so it is concerning.

**DR. CARTER-POKRAS:** Differential access and quality of care and implicit bias have been discussed in numerous reports over the past few decades, for example, with the 1985 Task Force Report on Black and Minority Health^13^ and the Unequal Treatment Report from the Institute of Medicine.^14^ After taking into account health insurance coverage and other issues, you still see that difference in access to certain treatments when you look at many cardiovascular and other health conditions.

The comment that Dr. Mouton just made is absolutely right. When it comes down to it during times of really tight constraints like what people are experiencing right now, decisions are indeed being made about who is going to receive care and who is not, and who is going to receive what could be considered lifesaving care and who is not. Unfortunately, racial and ethnic minorities are more likely to have conditions that put them at greater risk for severe complications. Right now, with COVID-19, underlying conditions that could prove to make outcomes worse could include diabetes, cardiovascular disease, hypertension, or asthma.^15^

We already know that racial and ethnic minorities have a disproportionate burden of illness and death.^16^ The ethicists certainly have their jobs cut out for them. Even though this is the first major pandemic we have had since 1918, it is not to say that we will not be dealing with another issue in the future wherein we are going to have to deal with major constraints and burdens to our existing health care facilities.

**DR. KIMMEL:** The mental health of our providers is highly stressed and strained. Important decisions are having to be made right now in almost a fight-or-flight mode, and those may tend to be even more likely wrought with implicit and unconscious bias. Because there is already so much bias against mental health, that is just adding another layer of complexity. As much as people are saying that we understand how stressful this is, I do not know that people always still really do fully understand. I just read an article on a Website about finding a therapist during this time, and it tries to give a number of resources no matter your insurance or need.^17^ However, our system is still very much biased against those needing mental health support and set up for self-pay. Although telehealth opens new avenues, it still is going to be more accessible for those who can afford to pay a therapist online through numerous services popping up to take advantage. Those who cannot afford these services will still have to deal with the same biases such as mental health care not always as covered as other health care needs. We are all bringing bias about what is needed and how to use resources appropriately.

***DR. FURR-HOLDEN:* We have talked a bit about telehealth and telemedicine. What do you think are some of the best practices that you think are being underutilized, and what do you see as really the stopgaps or policies that we need to implement as a part of the standard of care?**

**DR. MOUTON:** There is a major economic gap within the health care sector. Inner city and rural provider practice differ substantially in the financial resources they have available to reinvest in new practice technologies. I think that as we try to use technology to provide access to health care, without policy guidance and financial support, particularly from CMS, there will be a number of obstacles. If telehealth is here to stay, which most people say it is, then coupled with the expansion in digital health care monitoring, such as using the Internet of Things in people's homes to support better monitoring, we can start to see an intensification of the inequities for the communities served by these practices. What does that mean for someone who has no Internet and no things?

So, you talk about the Internet of Things, but many of our patients who are disadvantaged in terms of access and health care provision also lack those tools and devices. More and more, health care is going to move in that direction, and unless the funders of health care decide this is of big enough value to distribute to those who no longer can afford it or do not have it, to allow them to reap the benefits of this, we will again create a broader and deeper gap in terms of what people are able to receive and benefit from.

So, to me, that is something that, if we would address, would go a long way to bridging that gap. But it is going to take an investment to make that leap forward.

**DR. CARTER-POKRAS:** One of the things that has been brought to bear is that people are recognizing now how good or how poor their Internet access is in a way that I do not think they really fully understood previously.

We are understanding that in under-resourced communities where children have been expected to continue their schooling at home, they are unable to continue their online studying because they do not have enough bandwidth, even if they do have a computer.^18,19^ Schools have been making computers available to the children who do not have computers at home, but you have to stand in line within a certain period of time to go pick up the computers, and even then there may not be enough computers. And those who do finally get their hands on a computer are not given the online homework that they were supposed to have been given, or their parents cannot help them, whether it is because of language issues or other issues. So, in addition to universal health care, universal access to the Internet is something that we can put on our wish list.

On top of everything else, some people have been asked to vacate their housing.^20,21^ Can you imagine? Forget about access to the Internet, right? They have been asked to vacate their housing. They do not have a way to pay for their housing because they lost their job, and they have been asked to vacate their housing.

We are also seeing an increase in domestic violence^22^ and calls to mental health lines^23^ with people experiencing desperation, anxiety attacks, suicidal tendencies, et cetera, because of this. So universal health care and universal access to the Internet are two things we definitely want to think about.

In the meanwhile, we cannot have everything available only online. It has to be available in other ways that people can access because not everybody has access to the Internet right now.

***DR. FURR-HOLDEN:* It is interesting that you mention these points, like the mental health concerns and people being evicted, because again, we are thinking about, what are the policies to support some of these things? As an example, Michigan has issued a stay on all evictions, so people are not actually allowed to be evicted during this period. Now, I think about homeowners who maybe moved and are renting out their house but are dependent on that rental income to pay their mortgage. Well, their mortgage is still coming due, so all these pieces are connected. If you think about it from a policy perspective, I love when you say not just universal health care, but also universal access to the Internet, especially if we are talking about telehealth and telemedicine and some of these other things.**

**If we can identify the gaps, if we can see the problems, then how should we respond in terms of policy? What happened in Michigan is then they started adding to the stay of eviction order to prevent people from being evicted, because, of course, housing is so essential, if the whole order is to stay put to stay safe.**

**But then they started to put resources in place for noncommercial owners of homes who are providing housing so that they are also solvent during this time. Sometimes the one policy creates another problem that is just one step down the road. We need to be asking what these comprehensive policies are that will actually get us somewhere and create solutions, not displace or move the problems one tick down the road.**

**DR. KIMMEL:** Along those lines, I have talked to some pregnant women who already had housing instability before all this, and they had been saving up to get more stable housing of their own before the baby comes. But now they cannot go out and must continue to make difficult cramped housing situations continue to work. Many services individuals may have once accessed at a physical office, such as working with someone to get a housing voucher, cannot be done during this time when so many things have to remain closed. Online is now sometimes the only way to access things.

We need Internet, better Internet, but we also need better and more case management and services to help people navigate, because the system was always really fractured, but I feel like now it is even more unclear who can help and who cannot. When we are all allowed to go back to work, there are still going to be these downstream effects of when a person did not find housing at the critical time before the baby came.

***DR. FURR-HOLDEN:* I have what I call a “man-on-the-moon” idea. What I mean by that is, when they said “man on the moon,” that just seemed like it was purely aspirational and almost impossible to achieve at the time, but despite that, it was well resourced, and it happened. What do you see as the kind of the man on the moon? Where should we really be setting our sights if we are actually and truly going to deal with inequity in access to care?**

**DR. MOUTON:** I think because of the COVID-19 crisis, we now have a renewed focus on the need for an adequate public health infrastructure. I would like to see us set up a framework of a solid adequately funded public health and preventative care infrastructure that assures a baseline of health care access across all populations and all classes.

We couple that with key access provisions for communities across this country, both rural and urban, that gives access both to “brick and mortar” clinical practices as needed plus access to primary care, either by telehealth or in-person, which assures that these communities are able to maintain an adequate level of health that, in turn, allows the members of the community to enter the workforce, maintain income, and maintain social stability. That would be my moon shot.

**DR. CARTER-POKRAS:** I already talked about my wish for universal health care, but I would also stretch that to not having access to health care be tied to our workplaces because there are so many people who lost their jobs as a direct result of COVID-19, and that also means they lost their health insurance.

Health in all policies, also, I think that is really important.^24^ Health is created by a number of factors that go well beyond the scope of health care and public health activities. We gave the example about what happens when somebody gets evicted during this pandemic and the need for housing interventions at this time.^25^

I also think we need to have better appreciation for essential workers who have previously gone unnoticed. There are so many people who are keeping us going, whether it means our water coming through in our tap, or somebody picking up our garbage, or the people who are stocking the shelves at the grocery store, and hopefully providing them with the benefits that they need going forward. We could also benefit from having a better understanding of our supply chain, especially the food chain, and what we need to do to protect and maintain that.

***DR. FURR-HOLDEN:* I love that universal health care not being tied to work has been mentioned, and universal access to the Internet.**

**DR. CARTER-POKRAS:** And also, protecting our supply chain, especially our food chain, and protecting those who are essential employees, including those who up to this point many people did not even really see them. They were almost invisible in a sense.

**DR. KIMMEL:** All of these things are in line with what I would love to see. I think really trying to get people care in a way that meets their needs and is more individualized and personalized is a priority. For some people, that is going to mean being able to use the telephone to access care. For some people, that is going to mean that their Internet access should be improved. Then for some, these improvements still are not going to be the answer, so how can we also have safe places patients can go and use the Internet to do an appointment or that someone comes to them and takes them to a safe place?

To have this kind of individualized support will require thinking about how to expand our workforce for mental health support and use our workforce more effectively to ensure patients have time with supports and also those supports have time to personalize their approaches to the resources of each patient. This does not necessarily apply only health practitioners but also peer supporters and other community members who can be there to support mental health needs.

I grew up in a more rural region and now many of the patients I care for are from rural areas. We have so many rural families in North Carolina who have to drive hours to get to care. Is there a way that we can have someone who can go out to them and do a visit, who can bring the iPad to access their specialist? Can a psychiatrist provide supervision to a care manager who can go out to them and do that visit?

**DR. CARTER-POKRAS:** Thank you, Dr. Kimmel, for bringing that up, because our previous Surgeon General, Dr. Vivek Murthy, has really felt that loneliness is a crisis, an epidemic in our country. He is working on trying to get more information out about this crisis.^26^

I went through a 7-month training for a certificate in positive psychology and I am also a resilience trainer. It seems people are really desperate for finding ways to strengthen resilience skills now more than ever. In today's digital world, resilience is something that we would lift everybody's boats and help them identify how they can strengthen those social connections going forward.

And I just want to let you know, in addition to all of the other issues that I had mentioned earlier for my wish list, I would also add that language interpretation, improving transportation, and improving the ability to pay for care are also important. All of these things still need to be addressed in terms of improving access to care and quality of care.

**DR. FURR-HODEN:** Dr. Kimmel, you said something about expanding and increasing the health care workforce. I would be curious to hear your thoughts about how the public health infrastructure was not prepared to meet the demands of the COVID-19 pandemic. What are the immediate needs you see?

**DR. KIMMEL:** I think right now, so much of the focus is understandably on the immediate needs because so many people are getting seriously ill. And on top of that many health care workers are becoming ill and not able to come into work. With so much immediate need, we are focusing on the immediate needs and realizing the workforce does not have as much plasticity as we need.

But then even as COVID-19 cases slow and people are not getting so ill from the coronavirus, we are going to be left with large mental health needs. We will need to focus on building the workforce to cover those areas, too. We need to be building a workforce that can also help communities develop their resilience and help people build resilience.

Part of managing trauma is helping people realize how their stories are important, and what they have been through is important. This can help them see the strength that they have gotten from their experience. So we need that kind of workforce, as well, to build resilience. Building that kind of resilience requires time for people to sit down with people and talk, and it is hard to do that in a 15-min visit. How do you build a workforce wherein the work does not have to be done in that short of a visit? How do we have other people who can also fill that role?

**DR. MOUTON:** When I think about the workforce, I have kind of a contrarian view, particularly when you talk about the physician workforce. If we talk about the physician workforce, I think the issue substantially is a maldistribution problem more than an adequate numbers problem. And again, that is driven by economic factors and reimbursement issues rather than, I would argue, individual physician desires and practice hopes. I see economic factors related to where physicians choose to practice as a major driver.

Now, if we look at the health care workforce at large, I think we still have needs for many health professions in the context of COVID-19, particularly from nursing and respiratory therapy, to meet this acute need.

But I even wonder sometimes about that, because when you look at what is happening to the hospitals, due to the COVID-19-related economic forces, many of them are laying off nurses and looking at a future wherein there will be far fewer of these health care workers.

So, to me, it begs the question, if we have a true health care workforce shortage, why would economics be the sole driver in the decision to lay off a substantial workforce, because you still would need them to run your enterprise?

So, again, I wonder how much of this is driven by a distribution issue, and where people are being deployed, and where they are being located, as opposed to an absolute numbers issue. And it may be both.

***DR. FURR-HOLDEN:* If it is a distribution issue, how do we solve that problem? How do we build the kind of incentives so we can have people where the needs are versus where the resources are?**

**DR. MOUTON:** Well, right now the issue is that incentives are driven by the economic forces. As you move the economic forces out of the industry and say that we will deploy people where the need is the greatest, and they will be paid substantially the same or with some increased benefit for taking on those assignments, then that would be different.

Now, I am not saying that a universal one-payer health care system will necessarily do that, but it might. I have to study the distribution issues in Canada and in England to see how their systems work, but I think they are better prepared. People may argue about whether or not that is true. I think part of our issue is that economic factors drive where physicians live, practice, what specialty they choose, and what geographic area they live in. And without economics being the main motivating factor, figuring out how to distribute the health care workforce in a manner that takes that away, you could have a situation wherein people will choose to work in environments that give them better economic fulfillment.

***DR. FURR-HOLDEN:* Fantastic. This was an important conversation and I am glad you were all able to join me. Thank you all for participating, and for sharing your thoughts about what impacts equitable access to health care in the United States.**
